# Partial Deafness and Blindness Occurring in a Case of Pneumonitis

**Published:** 1840

**Authors:** T. J. Cogley

**Affiliations:** Mount Vernon, Ohio


Art. X.—*Partial Deafness and Blindness, occurring in a case of Pneumonitis.* By Dr. T. J. Cogley, of Mount Vernon, Ohio.

On the 15th February, I was called to see Mrs. W——, æt. twenty-eight years. I found her laboring under symptoms of pleuritis in the left side, which had been existing for twentyfour hours before I saw her. She was bled to incipient syncope with great relief; but in a short time reaction taking place, she was bled again to the same extent, which produced a decided reduction of the pain. She was then put upon tart. emet. in such doses as should nauseate considerably without producing emesis; but at first the medicine acted freely on the bowels. The use of it was continued with mucilaginous drinks, a cup of gruel occasionally, and, when required, a cathartic, for two or three days, without any decided amendment, except a partial reduction of the acute pain in the side, the difficulty of breathing, &c. About this time I examined the state of her lungs by percussion and auscultation. Finding the crepitant ronchus very distinct, I believed the inflammation to be now existing in the lungs as well as the pleura. The sputa had assumed the characteristic toughness of the first stage of pneumonia, the cough also had become much more troublesome, and the pain more obtuse and deeper seated. The pulse not seeming to admit of further bleeding, I cupped the thorax in the region in which the pain was felt, which somewhat moderated the pain, but it still existed so as to be troublesome. The pulse gradually became weaker, until it was nearly extinct; and now (about the sixth day) the patient began to complain of hardness of hearing and dimness of vision. The antimony was continued regularly, and the bowels moved by calomel, followed by salts or oil when required. After cupping the chest, a large epispastic was applied over the seat of the pain. The deafness, as well as derangement of vision, increased to an extent which very much alarmed her friends. It was now with the utmost difficulty that the patient could be made to hear the questions which were asked in relation to her feelings; at the same time she could not distinguish between individuals. From the time these symptoms commenced, the patient became slightly comatose. Auscultation, percussion and the sputa, indicated that the inflammation was extending in the left lung. I think the right lung remained free from organic disease during the whole course of the affection. The left lung was now evidently hepatized to a considerable extent. The percussion being perfectly flat, and the respiratory murmur in at least all the lower half of the lung, completely extinguished. It was not a fluid in the cavity of the pleura that produced the dullness on percussion, for its boundaries were the same in the erect and recumbent posture. The pain was not very troublesome now, but still existed. The patient was drowsy at all times, but did not sleep, and could scarcely be got to hear at all, and could not distinguish any object, though she could discern the light. She could not bear a candle to be brought into the room.

I persevered in the exhibition of ant. tart., in conjunction with calomel and Dover's powder; kept up counter-irritation by repeatedly blistering over the seat of the pain; gave a solution of cream of tartar in mucilage as a drink, and for the purpose of increasing the secretion of urine. As soon as the gums became slightly swollen, the secretion of urine was greatly increased, the hardness of hearing and derangement of vision began to subside, and by the fourteenth day of the disease the patient could hear tolerably well; and by the eighteenth or twentieth, both hearing and seeing were perfectly restored. Convalescence was extremely tardy, as it was also in two other cases of the same description that came under my notice. A slight dulness on percussion still exists, but is gradually subsiding.

If the above is deemed worthy of notice, I would be much pleased to see the opinions of able men on the subject. Do such cases frequently occur, and is it pathognomic of any particular pathological condition of the lungs? are questions I should like to hear answered. I have never read any thing explanatory of such symptoms, nor have I heard any practitioner speak of such cases.

*March,* 1840.

